# Using Goldmann Visual Field Volume to Track Disease Progression in Choroideremia

**DOI:** 10.1016/j.xops.2023.100397

**Published:** 2023-09-14

**Authors:** Adam P. DeLuca, S. Scott Whitmore, Nicole J. Tatro, Jeaneen L. Andorf, Ben P. Faga, Laurel A. Faga, Malia M. Colins, Meagan A. Luse, Beau J. Fenner, Edwin M. Stone, Todd E. Scheetz

**Affiliations:** 1 The University of Iowa Institute for Vision Research & Department of Ophthalmology and Visual Sciences, Carver College of Medicine, The University of Iowa, Iowa City, Iowa; 2 Department of Medical Retina, Singapore National Eye Centre, Singapore; 3 Singapore Eye Research Institute, Singapore; 4 Ophthalmology and Visual Sciences Academic Clinical Programme, SingHealth Duke-NUS Academic Medical Centre, Duke-NUS Graduate Medical School, Singapore

**Keywords:** Goldmann kinetic perimetry, Visual fields, Choroideremia, Inherited retinal disease

## Abstract

**Purpose:**

Choroideremia is an X-linked choroidopathy caused by pathogenic variants in the *CHM* gene. It is characterized by the early appearance of multiple scotomas in the peripheral visual field that spread and coalesce, usually sparing central vision until late in the disease. These features make quantitative monitoring of visual decline particularly challenging. Here, we describe a novel computational approach to convert Goldmann visual field (GVF) data into quantitative volumetric measurements. With this approach, we analyzed visual field loss in a longitudinal, retrospective cohort of patients with choroideremia.

**Design:**

Single-center, retrospective, cohort study.

**Participants:**

We analyzed data from 238 clinic visits of 56 molecularly-confirmed male patients with choroideremia from 41 families (range, 1–27 visits per patient). Patients had a median follow up of 4 years (range, 0–56 years) with an age range of 5 to 76 years at the time of their visits.

**Methods:**

Clinical data from molecularly-confirmed patients with choroideremia, including GVF data, were included for analysis. Goldmann visual field records were traced using a tablet-based application, and the 3-dimensional hill of vision was interpolated for each trace. This procedure allowed quantification of visual field loss from data collected over decades with differing protocols, including different or incomplete isopters. Visual acuity (VA) data were collected and converted to logarithm of the minimum angle of resolution values. A delayed exponential mixed-effects model was used to evaluate the loss of visual field volume over time.

**Main Outcome Measures:**

Visual acuity and GVF volume.

**Results:**

The estimated mean age at disease onset was 12.6 years (standard deviation, 9.1 years; 95% quantile interval, 6.5–36.4 years). The mean field volume loss was 6.8% per year (standard deviation, 4.5%; 95% quantile interval, 1.9%–18.8%) based on exponential modeling. Field volume was more strongly correlated between eyes (*r*^2^ = 0.935) than best-corrected VA (*r*^2^ = 0.285).

**Conclusions:**

Volumetric analysis of GVF data enabled quantification of peripheral visual function in patients with choroideremia and evaluation of disease progression. The methods presented here may facilitate the analysis of historical GVF data from patients with inherited retinal disease and other diseases associated with visual field loss. This work informs the creation of appropriate outcome measures in choroideremia therapeutic trials, particularly in trial designs.

**Financial Disclosure(s):**

Proprietary or commercial disclosure may be found in the Footnotes and Disclosures at the end of this article.

Visual decline in progressive inherited retinal disease typically follows one of several patterns, with the 2 most common patterns being early loss of peripheral vision in retinitis pigmentosa and early loss of central vision in cone and macular dystrophies. These patterns of vision loss inform the appropriate therapeutic approaches that might be taken for the development of gene- or cell-based therapies. Unlike these patterns, choroideremia, an X-linked choroidopathy caused by pathogenic variants in the *CHM* gene,^1^, ^2^, ^3^, ^4^, ^5^, ^6^ is peculiar in that vision loss typically begins with the appearance of multiple peripheral areas of chorioretinal atrophy that spread centripetally and coalesce, usually sparing the foveal center until late in the disease.^7^^,^^8^ This progression pattern makes quantitative monitoring of the decline in visual function particularly challenging in patients with choroideremia.

Choroideremia is an attractive target for gene therapy. Its relatively small cDNA size is favorable for packaging into a variety of gene delivery vectors, and the loss-of-function nature of most disease-causing variations in the gene is more suitable for a gene augmentation strategy than diseases caused by dominant gain-of-function mutations. Despite these favorable features, the development of effective treatments for choroideremia has been hampered by the relative unpredictability of visual acuity (VA) loss. When the fovea of 1 eye becomes affected by the disease, VA is rapidly lost but is often preserved in the fellow eye for many years.^8^, ^9^, ^10^, ^11^ This intereye variability is further complicated by interpatient variability, even between individuals with matching genotypes.^9^^,^^12^^,^^13^ The variability in VA outcomes in a seminal 2014 choroideremia phase I/II trial of gene therapy^14^^,^^15^ and in a more recent trial^16^ suggests that alternative outcome measures may be more relevant for future trials. Alternative methods of progression monitoring, including autofluorescence and swept-source OCT, are highly sensitive and reproducible^17^, ^18^, ^19^ but do not directly measure visual function, particularly in the peripheral islands of vision that can form an important part of preserved vision in patients with choroideremia. Conventional methods of visual field assessment such as Goldmann kinetic perimetry provide a more complete representation of the visual experience of a patient and are acceptable as a clinical trial outcome measure. However, in choroideremia, the pattern of field loss is highly heterogeneous between patients,^20^^,^^21^ and quantitative comparison between eyes or between patients is challenging.

To address this challenge, we report the development of a novel method for the quantitative assessment of Goldmann kinetic perimetry outcomes in a cohort of patients with choroideremia with long-term follow-up. We show that this method enables reproducible quantification of the visual field volume, even for patients with highly complex patterns of visual field loss. Using this method, we demonstrate progressive loss of the visual field over long follow-up periods with minimal intereye variability. Visual field volume is contrasted with VA loss in patients with choroideremia, which shows slow progression early in the disease and relatively rapid loss of acuity late in the disease, producing high intereye variability. We applied a mixed-effects model to these visual field volume data to predict vision outcomes relevant to therapeutic clinical trials for choroideremia.

## Methods

### Study Cohort

This was a retrospective cohort study of patients with choroideremia seen at the University of Iowa Department of Ophthalmology and Visual Sciences from 1966 to 2023. We included male patients with molecularly-confirmed pathogenic variants in the *CHM* gene. Female manifesting carriers were excluded from this study because random X-inactivation causes a different pattern of disease in these patients. All patients consented to the research study, which was approved by the University of Iowa Institutional Review Board. The study was conducted in accordance with the tenets of the Declaration of Helsinki.

### Clinical Examination

All patients underwent a comprehensive clinical examination, including a detailed history of illness, pedigree review, subjective refraction, and slit lamp biomicroscopy. Goldmann visual field (GVF) data were recorded with a Goldmann kinetic perimeter (Haag-Streit). Eye charts for VA tests varied over the study period, so all acuities were converted to logarithm of the minimum angle of resolution by modifying the procedure used by Tiew et al^22^ to account for the number of optotypes present on each line of the chart. When the chart type was not recorded, it was assumed to be the type most used at the time.

### Genotyping

Blood samples were obtained from patients with a clinical diagnosis of choroideremia, genomic DNA was extracted, and Sanger sequencing of the *CHM* gene was performed using previously described methods.^23^ For 10 cases in which large copy number variants were suspected, low coverage (15×) whole genome sequencing was used to confirm the variant and identify breakpoints. These breakpoints were confirmed using Sanger sequencing in the probands and available family members.

### Visual Field Data Extraction

**Figure 1:**
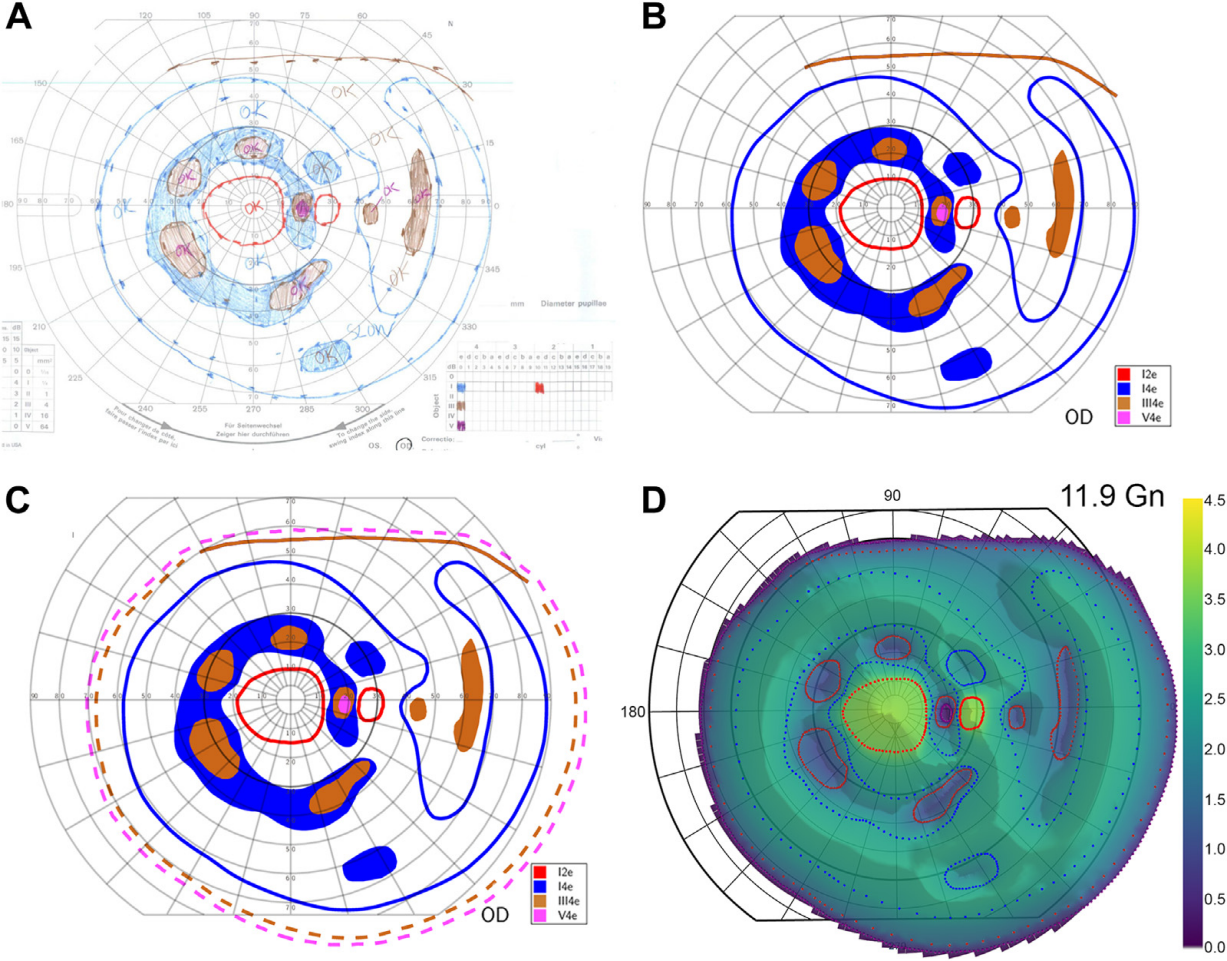
Steps in processing Goldmann visual fields to compute visual field volume. **A,** The Goldmann visual field record is digitized. **B,** Isopters are traced using TruthMarker, an annotation software for the iPad. **C,** Isopters incompletely traced by perimetrists are completed computationally, and any crossing isopters are uncrossed. **D,** The hill of vision is constructed by interpolating across isopters. The data shown in this example are from patient P3. Gn = Goldmann unit; OD = right eye.

Goldmann perimetry was performed at the University of Iowa using a subset of the I1e, I2e, I3e, I4e, III4e, and V4e targets. Paper records were scanned (Fig 1A) and traced using an iPad-based annotation system (TruthMarker, The University of Iowa Institute for Vision Research).^24^ This software collects (1) splines representing isopters, (2) fiducial points along the horizontal and vertical axes, (3) the laterality of the field, (4) the date of visit, and (5) the set of isopters tested. These data were used to render the traced field (see Fig 1B) with all identifying and extraneous marks removed. Renderings were overlaid onto the original clinical data to identify and resolve any inconsistencies, thereby ensuring an accurate reproduction of the original field. To validate our tracing procedure, we traced a set of fields in duplicate, and independent tracers produced nearly identical results (*r*^2^ = 0.9999). This was consistent with previous reports.^25^

### Standardizing Data

Because GVF data were collected over decades as part of clinical care, the isopters assessed varied between tests, and some isopters were incomplete. We took 4 steps to address these challenges: (1) we excluded fields conducted for nonclinical purposes, such as driving licenses, fields halted due to clinic demand, fields that did not evaluate at least the I2e and I4e targets, and fields that were not physiologically possible. (2) We completed open isopters using the shape of the next smaller/dimmer isopter as a guide. First, we measured the distances between the ends of the open isopter and the closest points on the guide isopter. Next, for each point between these closest points, we calculated the distance to the interpolated curve by linearly interpolating the distance between the ends of the incomplete isopter and the guide isopter. Finally, we projected an interpolated point at that distance perpendicular to the guide isopter. This procedure connects the ends of an open isopter by a curve that follows the shape of the guide in the region that was not measured. (3) We performed a similar procedure using normative data^26^ as isopter guides for fields where V4e and possibly III4e targets were not tested due to the patient having a relatively normal peripheral field. In field renderings such as in Figure 1C, we denoted interpolated/extrapolated isopters or parts thereof with dashed lines. Automated interpolations/extrapolations were manually reviewed, and inconsistencies were corrected. (4) In patients with hand motion vision or worse, VA was recorded, but perimetry was frequently not performed. This was likely done to save time by not performing a clinically unnecessary test, but it creates a bias toward nonzero field volumes. To avoid this bias, we assumed GVF volume to be zero in these cases, involving 30 fields from 9 patients.

### Modeling the Hill of Vision

The 3-dimensional (3D) hill of vision is important in quantifying vision and visualizing kinetic perimetry data. We used the target luminance (log_10_ [10^3^ {cd/m^2^}^−1^]) of the commonly collected isopters (I1e, I2e, I3e, I4e, III4e, and V4e), as calculated by Christoforidis,^27^ as the “height” of the 3D hill of vision. To determine the height of regions within the hill of vision, a linear interpolation methodology was used.

First, the organization of the isopters was modeled as a graph structure, in which isopters contained within another isopter were denoted as children of the containing isopter. Each parent isopter may contain multiple child isopters. The relationship graph was trimmed such that only those parent–child relationships that were the closest bounds were retained. Thus, in the case of a I2e inside a I3e and a I4e, the I2e is the child of the I3e, and the I3e is the child of the I4e, but the I2e is not the child of the I4e. We refer to this graph structure of isopter relationships as the hierarchy of structure.

For each isopter, a skeletonization operation was used to identify a midline between isopter boundaries. To compute the skeleton for a given isopter, the areas encompassed by any child isopters and scotomas were first removed from the bounding isopter’s area. The resulting polygon was skeletonized using scikit-image.^28^ The height of the skeleton was determined based upon the bounding isopter. For cases in which the bounding isopter was a standard isopter (i.e., not a scotoma), the height was set to halfway between the bounding isopter and the next higher isopter from the set of commonly collected isopters. For the I1e isopter, the skeleton height was set to 4.25. For cases in which the bounding isopter was a scotoma, the height of the skeleton was set to halfway between the bounding isopter and the next lower isopter from the set of commonly collected isopters.

Next, to model the shape of the hill of vision, points were evaluated using the polar coordinate system in steps of 2 degrees, with theta varying from 0 to 360 degrees and the radius varying from 0 to 100 degrees. For each point, using the hierarchy of structure, the bounding isopter was identified. The bounding isopter is the isopter containing the point, such that none of its child isopters contains the point. The isopter nearest to the point was determined from the set consisting of the bounding isopter and its children. The height of the point was then computed as a weighted average of the heights of the skeleton and the nearest isopter, in which the weights were inversely proportional to the distance to the skeleton and the nearest isopter. Thus, the closer isopter or skeleton contributes more to the overall height. If the given point did not fall within any isopter, then the height at that point was set to zero. This 3D model of the hill of vision was then graphed as a Mesh3D object in Plotly (open-source software package) (Fig 1D).

Finally, to calculate the volume of the hill of vision, the visual field was divided into a grid of small regions. The height for each point, modeled above, was multiplied by the solid angle in steradians to correct for the cartographic error present in the planar representation.^29^ The resulting units of volume are referred to as Goldmanns, abbreviated Gn.^27^ We validated the area calculation by comparing a set of fields on our platform and on Weleber’s VisFields program (version 9/25/1991).^29^ The 2 programs produced nearly identical results (*r*^*2*^ = 0.9995).

### Statistical Modeling for Disease Progression

The retrospective nature of the dataset and the range of possible volume values posed 3 challenges to statistical modeling: (1) past studies indicate that the progression of choroideremia is nonlinear, following an exponential decay function over time,^30^^,^^31^ but it remains unclear if the onset of exponential decline occurs at birth or later.^8^^,^^11^ (2) The number and timing of available visual fields vary widely between patients. (3) Goldmann perimetry can record GVF volumes that are ≥ 0. Thus, the likelihood of the data does not follow a normal distribution.

To address these challenges, we fit a Bayesian mixed-effects hurdle model to the GVF volumes using rethinking^32^ (version 2.21; https://github.com/rmcelreath/rethinking), a software package for the R programming language^33^ (version 4.1.3, open-source software package). The rethinking package provides a convenient interface to the Stan programming language for Bayesian modeling^34^ (version 2.26.13, open-source software packages). We built a single model from 2 components. The first component (equation 1) is a delayed exponential function, with a normal likelihood and mixed effects for delay (i.e., age of onset) and the rate of progression. This component addresses challenges 1 and 2. The second component (equation 2) is a “hurdle,” modeling the probability of observing a volume of 0. This component addresses challenge 3. Combined, the probability of the data is given by 2 formulas:

(1) Pr(V|V>0)=(1−θ)×Normal(μ,σ)

(2) Pr(V|V>0)=θ

The full model and choice of priors is described in Supplemental Methods (available at https://www.ophthalmologyscience.org).

### Code and Data Availability

The TruthMarker iPad app that was used for tracing the visual fields is available for free on the Apple App store (https://apps.apple.com/us/app/truthmarker/id1525749443). Instructions on its configuration and use are available at https://ivr.uiowa.edu/TruthMarker. The hill of vision modeling software is available at https://github.com/scheetzt/VisualFieldAnalysis. The code and data used in this manuscript are available at https://github.com/barefootbiology/chmgvf2023.

## Results

### Patient Characteristics

A total of 238 clinic visits of 56 molecularly-confirmed male patients with choroideremia were included in the study. Of total, 338 (30 assumed zero) visual fields were obtained from 52 of 56 of these patients. The pathogenic variants we detected in this cohort are shown in Table 1, with the full Human Genome Variation Society specification for each variation in Table S2 (available at https://www.ophthalmologyscience.org). The median age at their first visit to our clinic was 30 (range, 5–73), and the median follow-up duration was 4 years (range, 0–56). The median VA at presentation was 0.089 logarithm of the minimum angle of resolution units (range, −0.161 to 2.7). Fundus findings were typical of choroideremia and ranged from peripheral areas of depigmentation of the retinal pigment epithelium with choroidal thinning to extensive chorioretinal atrophy involving the fovea. *CHM* genotypes were most commonly deletions or stop mutations, with Arg253Stop being the most common single pathogenic variant (14/56 patients and 7/41 families).

**Table 1:** Genotypes of the Choroideremia Study Cohort

IND	FAM	Mutation	cDNA/Genomic
P1	F1	Deletion of 7 genes∗	Breakpoint not determined
P2	F1	Deletion of 7 genes∗	Breakpoint not determined
P3	F2	Deletion of exon 15	Breakpoint not determined
P4	F3	Deletion of exons 9–11	Breakpoint not determined
P5	F4	Deletion exons 3–8	Breakpoint not determined
P6	F5	Deletion of exons 1–2	chrX:85244832-85471922del
P7	F6	Complex genomic variation†	**-**
P8	F7	His402 del5cATTCA ins3TTC	c.1205_1209delATTCAinsTTC
P9	F8	Deletion of entire CHM gene	chrX:84539284-86055979del insTGCCCCA
P10	F9	Deletion of entire CHM gene	chrX:84790935-87522740del insTATA
P11	F9	Deletion of entire CHM gene	chrX:84790935-87522740del insTATA
P12	F10	Deletion of entire CHM gene	chrX:85100378-85626665del
P13	F11	Deletion of exons 9–15	chrX:85100822-85178695del insTATTCAATCTCATTATTCATTATTGTTCATTAT
P14	F12	Leu527 del4aaaTTGT	c.1579_1582delTTGT
P15	F13	Leu527 del4aaaTTGT	c.1579_1582delTTGT
P16	F14	Deletion of exon 12	chrX:85145877-85152933del
P17	F14	Deletion of exon 12	chrX:85145877-85152933del
P18	F15	Ser453 del2tCCins1tG	c.1358_1359delCCinsG
P19	F16	Gln448Stop CAA>TAA	c.1342C>T
P20	F17	Ser445Stop TCA>TGA	c.1334C>G
P21	F18	Deletion of exons 1–8	chrX:85203106-85546828del
P22	F18	Deletion of exons 1–8	chrX:85203106-85546828del
P23	F19	Duplication exons 6–8	chrX:85204844-85215309dup
P24	F19	Duplication exons 6–8	chrX:85204844-85215309dup
P25	F20	Leu365 del2cTT	c.1094_1095delTT
P26	F21	Thr300ins1cttA	c.897_898insA
P27	F22	Arg293Stop CGA>TGA	c.877C>T
P28	F23	Met289 ins2aTG	c.865_866insTG
P29	F24	Phe257 del2gagTT	c.769_770delTT
P30	F25	Arg253Stop CGA>TGA	c.757C>T
P31	F26	Arg253Stop CGA>TGA	c.757C>T
P32	F27	Arg253Stop CGA>TGA	c.757C>T
P33	F27	Arg253Stop CGA>TGA	c.757C>T
P34	F28	Arg253Stop CGA>TGA	c.757C>T
P35	F28	Arg253Stop CGA>TGA	c.757C>T
P36	F28	Arg253Stop CGA>TGA	c.757C>T
P37	F28	Arg253Stop CGA>TGA	c.757C>T
P38	F28	Arg253Stop CGA>TGA	c.757C>T
P39	F28	Arg253Stop CGA>TGA	c.757C>T
P40	F28	Arg253Stop CGA>TGA	c.757C>T
P41	F29	Arg253Stop CGA>TGA	c.757C>T
P42	F30	Arg253Stop CGA>TGA	c.757C>T
P43	F31	Arg253Stop CGA>TGA	c.757C>T
P44	F32	Arg239Stop CGA>TGA	c.715C>T
P45	F33	Ile215 del4aTTAC	c.644_647delTTAC
P46	F34	Thr175 del2acAG	c.525_526delAG
P47	F35	Thr175 del2acAG	c.525_526delAG
P48	F35	Thr175 del2acAG	c.525_526delAG
P49	F36	IVS4-2del4 tAGTC	c.315-2_316delAGTC
P50	F36	IVS4-2del4 tAGTC	c.315-2_316delAGTC
P51	F36	IVS4-2del4 tAGTC	c.315-2_316delAGTC
P52	F37	Gln106Stop CAG>TAG	c.316C>T
P53	F38	212.3kb Insertion‡	**-**
P54	F39	IVS2+1 G>A	c.116+1G>A
P55	F40	IVS1+1 G>T	c.49+1G>T
P56	F41	Ser7 del12tcGGAGTTTGATGT	c.21_32delGGAGTTTGATGT

FAM = family ID; IND = individual ID.

The genotype for each patient in the cohort is presented. All variations are in the context of the hg19 genome build (NC_000023.10) and using the current reference sequence for the *CHM* transcript (NM_000390.4).

∗ Deletion of 7 genes detected by chromosomal microarray: LOC101928128, ZNF711, POF1B, MIR1321, MIR361, CHM, and DACH2.

† 87.2 kb deletion including CHM exon 15 followed by an 8661 bp insertion consisting of an inverted CHM exon 15 and these additional base pairs: CTCAATTGCAAATG.

‡ Alu-mediated 212.3 kb insertion of a fragment of chromosome 3 (approximately chr3:121534573-121746886) into intron 2 of CHM (approximately chrX:85267479-85267494); the chr3 insertion is flanked by a 32 bp insertion of GGCCGGGCGCGGTGGCTCACGCCTGTAATCCC and a 20 bp insertion of TGGAAGTTAAGTTAGTTACT.

### Conversion of GVFs to Volumetric Data in Patients with Choroideremia

Visual fields of the patients with choroideremia varied widely, often containing multiple scotomas, peripheral defects, and islands of preserved vision (Fig 1A). We scanned and manually traced each field, producing 1:1 digital representations of the fields (Fig 1B) with missing isopters added by interpolation (Fig 1C). Topographical representations of the hill of vision were generated (Fig 1D), and from these representations, the visual field volume was computed.

### Visual Field Outcomes in Patients with Choroideremia

**Figure 2:**
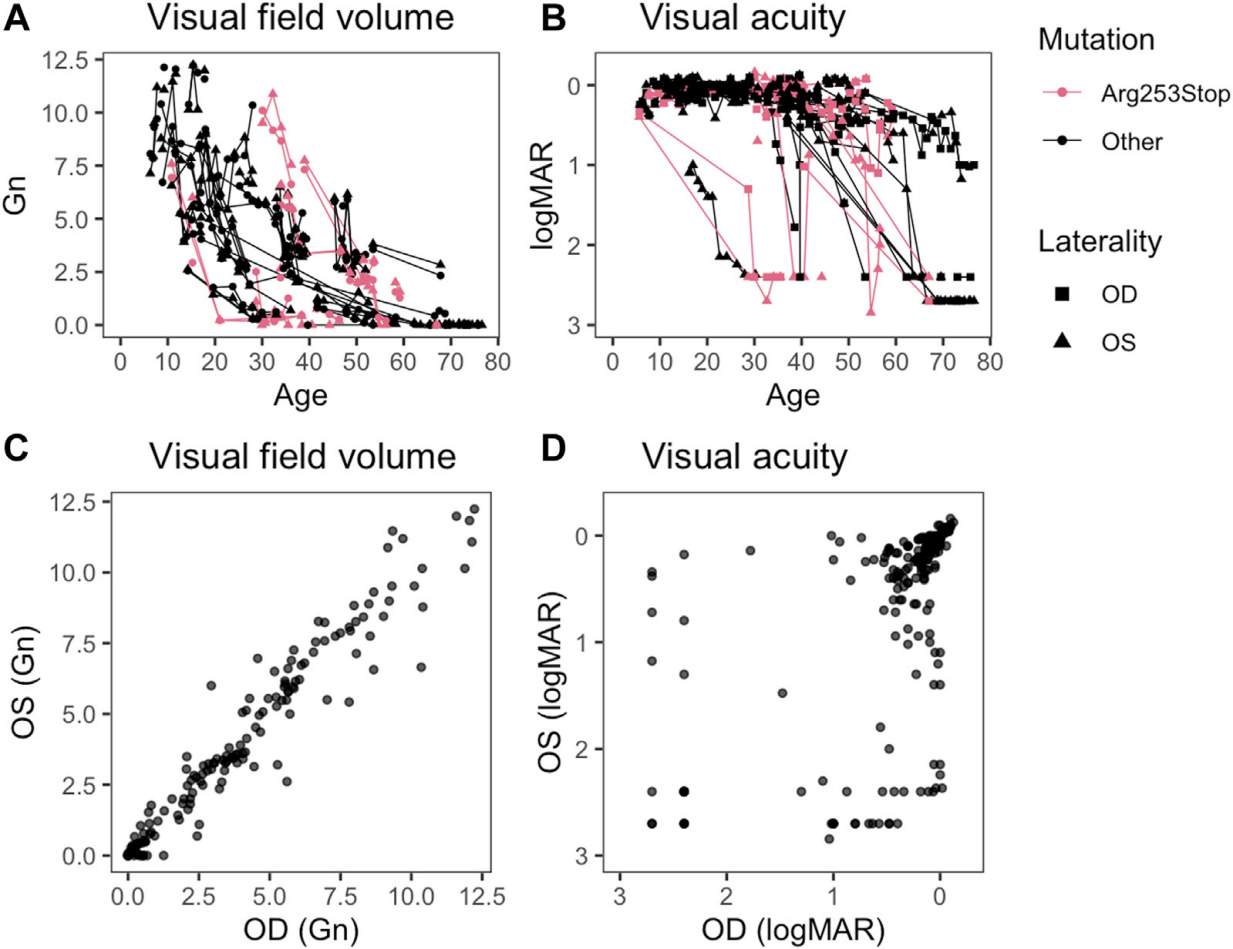
Visual field and visual acuity outcomes in the choroideremia cohort. **A,** Visual field volume declines with age, and apparent age of onset varies by decades between individuals. **B,** Visual acuity shows stable vision in most eyes for the first 4 decades followed by precipitous decline, often in a single eye. **C,** Visual field volumes of the left and right eyes are highly correlated (*r*^2^ = 0.935), whereas (**D**) visual acuities of the left and right eyes are poorly correlated (*r*^2^ = 0.285), due to variable age of foveal loss between eyes. Gn = Goldmann unit; logMAR = logarithm of the minimum angle of resolution; OD = right eye; OS = left eye.

**Figure 3:**
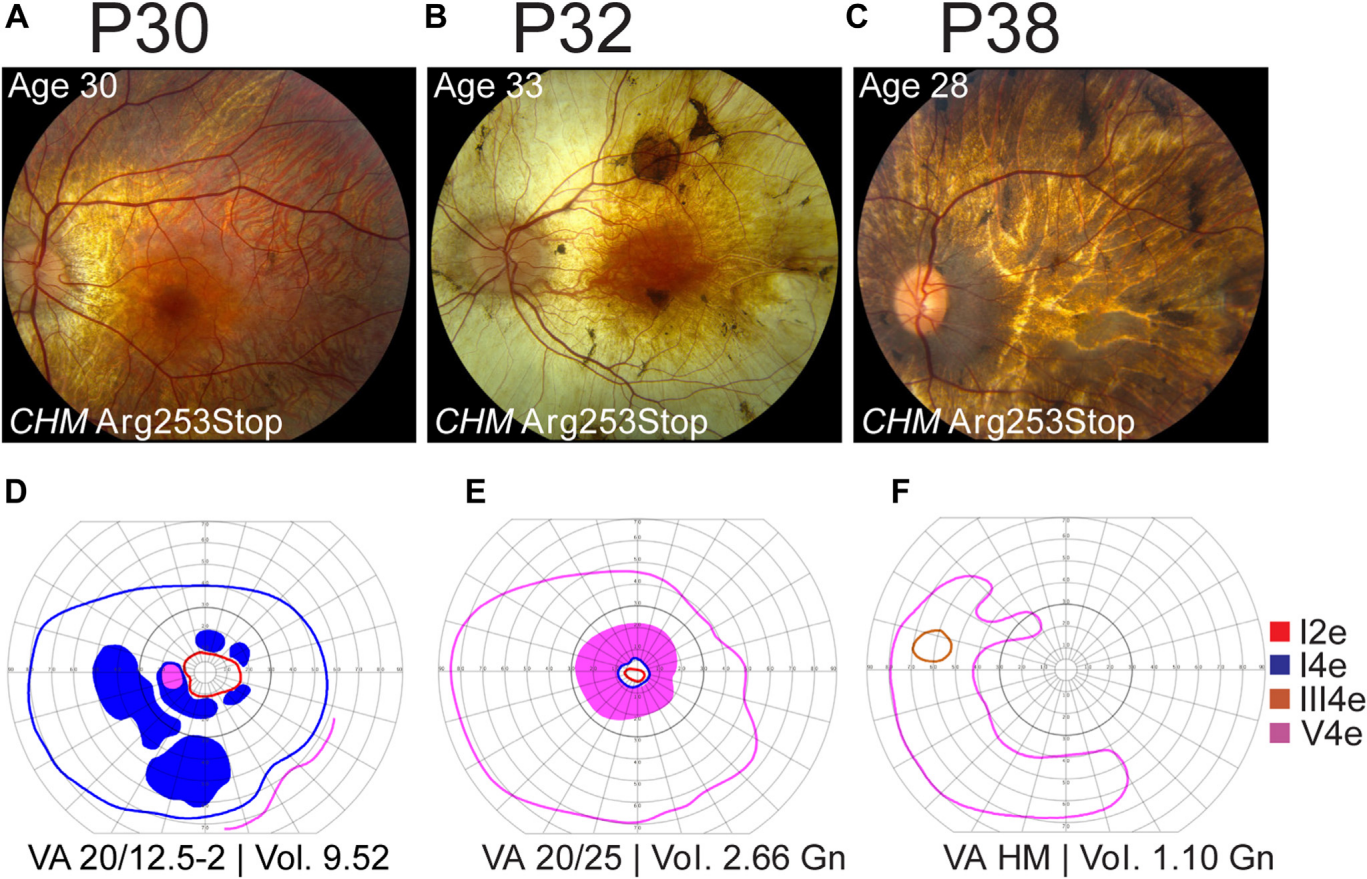
Structural and functional discordance in genotype-matched patients with choroideremia. Fundus photographs of the left eye from 3 individuals of similar age with a *CHM* Arg253stop variant are shown in panels (**A**–**C**). The corresponding traced Goldmann visual fields are shown in panels (**D**–**F**). The visual acuity (VA) and calculated visual field volumes (Goldmann units, Gn) at the time of visual field assessment are shown below. HM = hand motions.

Longitudinal visual field volume and VA outcomes are shown in Figure 2. Visual field volume showed variable rates of decline, with 12 patients reaching perimetric blindness (Gn = 0) between the ages of 29 and 73 (Fig 2A). With several exceptions (n = 6), VA generally remained > 0.5 logarithm of the minimum angle of resolution units (20/63 Snellen units) until age 40, after which there was either a slow or rapid decline of either eye (Fig 2B). We did not observe any genotype–phenotype correlations in these data. The most common disease-causing genotype in this cohort was Arg253Stop. Patients with this genotype (Fig 2A, B, shown in red) were among the most and least severely affected patients in the cohort and had remarkably different phenotypes at similar ages (Fig 3). Intereye correlation of field volume was high (*r*^2^ = 0.935; Fig 2C), while VA was poorly correlated between eyes (*r*^2^ = 0.285; Fig 2D).

**Figure 4:**
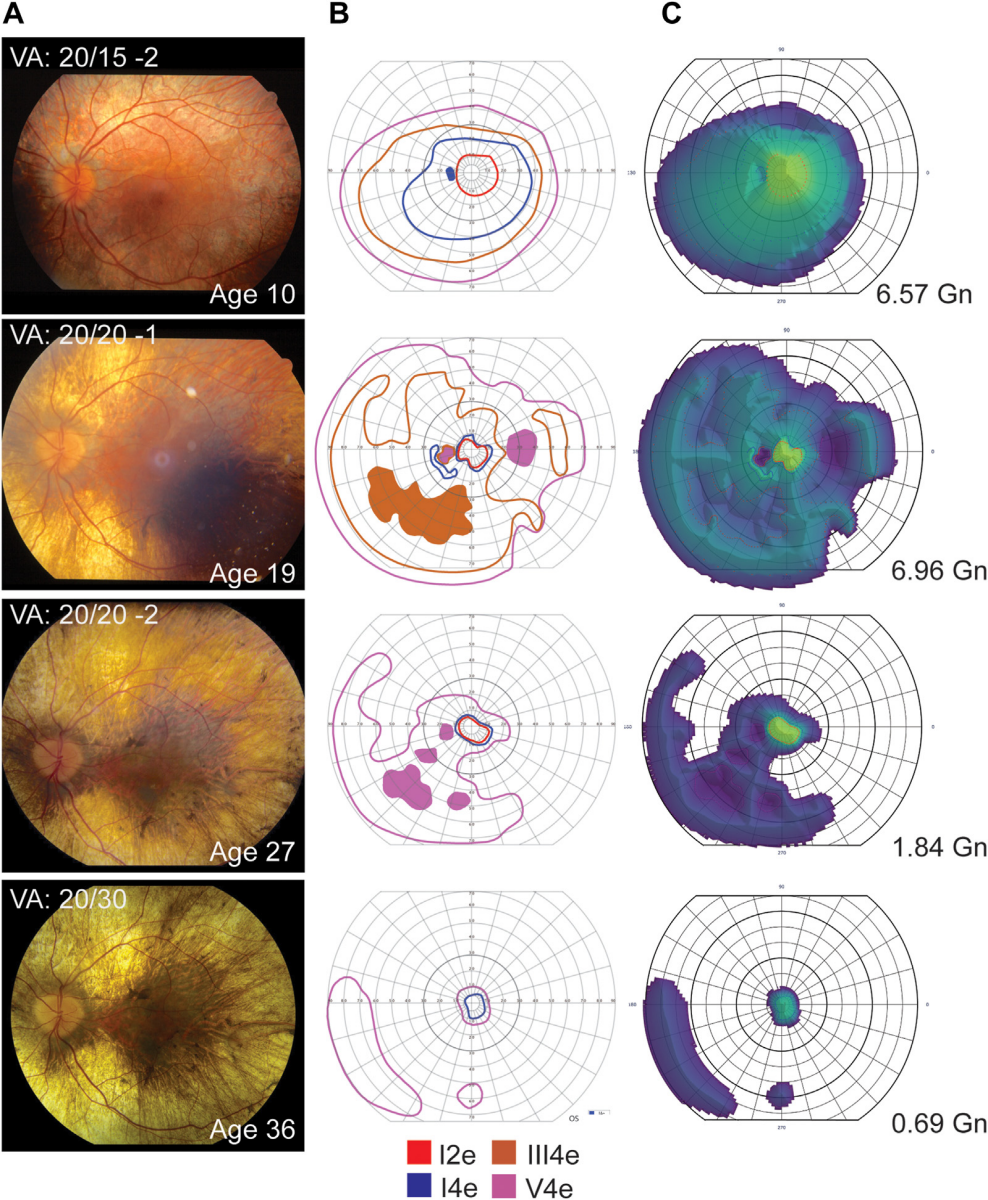
Disease progression in an individual with choroideremia. Changes in fundus appearance (**A**), traced Goldmann visual fields (**B**), and estimated visual field volumes (**C**) over a 26-year follow-up period are shown for the left eye. Field volume measurements are shown in Goldmanns (Gn) for each field. The patient (P56) had molecularly-confirmed choroideremia with a *CHM* Ser7del12tcGGAGTTTGATGT genotype. VA = visual acuity.

A representative longitudinal series of fundus images with corresponding visual fields and field topograms for a patient is shown in Figure 4. Progressive chorioretinal atrophy seen on fundus photography coincided with constriction of the visual field and reduced visual field volume, declining from 6.57 Gn to 0.69 Gn over the course of 25 years.

### Visual Field Volume is Robust to Inconsistencies in a Retrospective Dataset

**Figure 5:**
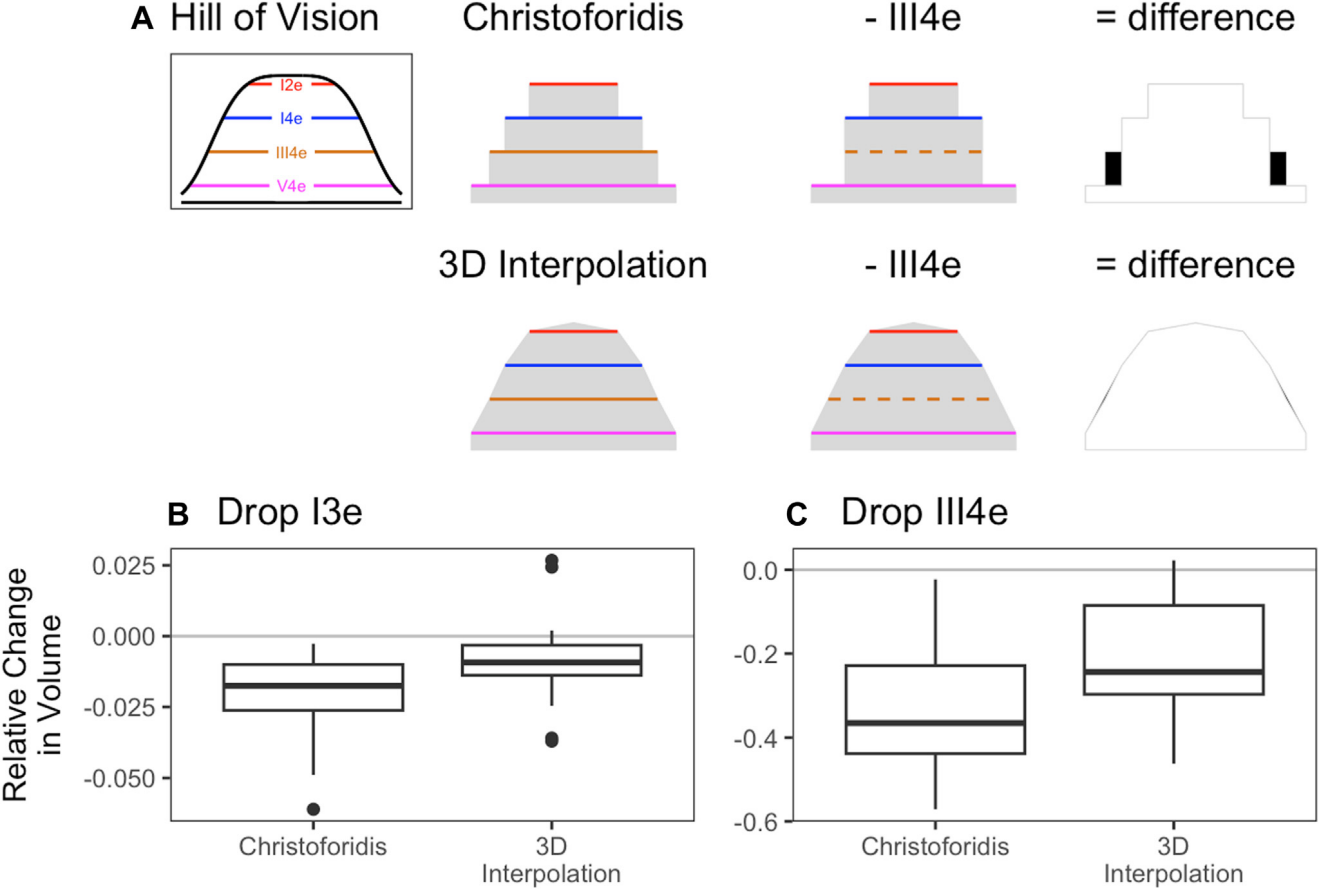
A comparison of the loss of volume in the presence of missing data. **A,** Idealized model illustrating the procedure for computing volume with and without the III4e isopter for Christofordis' stepwise method and for our method. **B-** The interpolation model presented in this manuscript is compared to the stepwise method. This was performed by (**B**) removing the I3e isopter or the (**C**) III4e isopter from each field, recomputing the volume, and comparing the relative loss. 3D = 3-dimensional.

Visual field assessment is subjective and prone to inconsistencies such as missing or incomplete isopters and variable patient or perimetrist performance. Robustness of the interpolation technique to missing isopters is demonstrated in Figure 5, in which an existing isopter is withheld. The loss of volume is compared between the interpolated model described in this manuscript and the stepwise 3D form described by Christoforidis.^27^ In both the case of interpolating the I3e and III4e isopters, the 3D interpolation method outperforms the assumption that a larger or brighter target will be seen at least in the same area as the smaller or dimmer target. It should be noted that because the largest/brightest targets are not typically tested in normal fields, the set of fields we are evaluating here would be enriched for more complex visual fields. This would likely result in an overestimation of the true error of our interpolation method.

### Volume Measurements Show a Wider Dynamic Range than Individual Isopters

**Figure 6:**
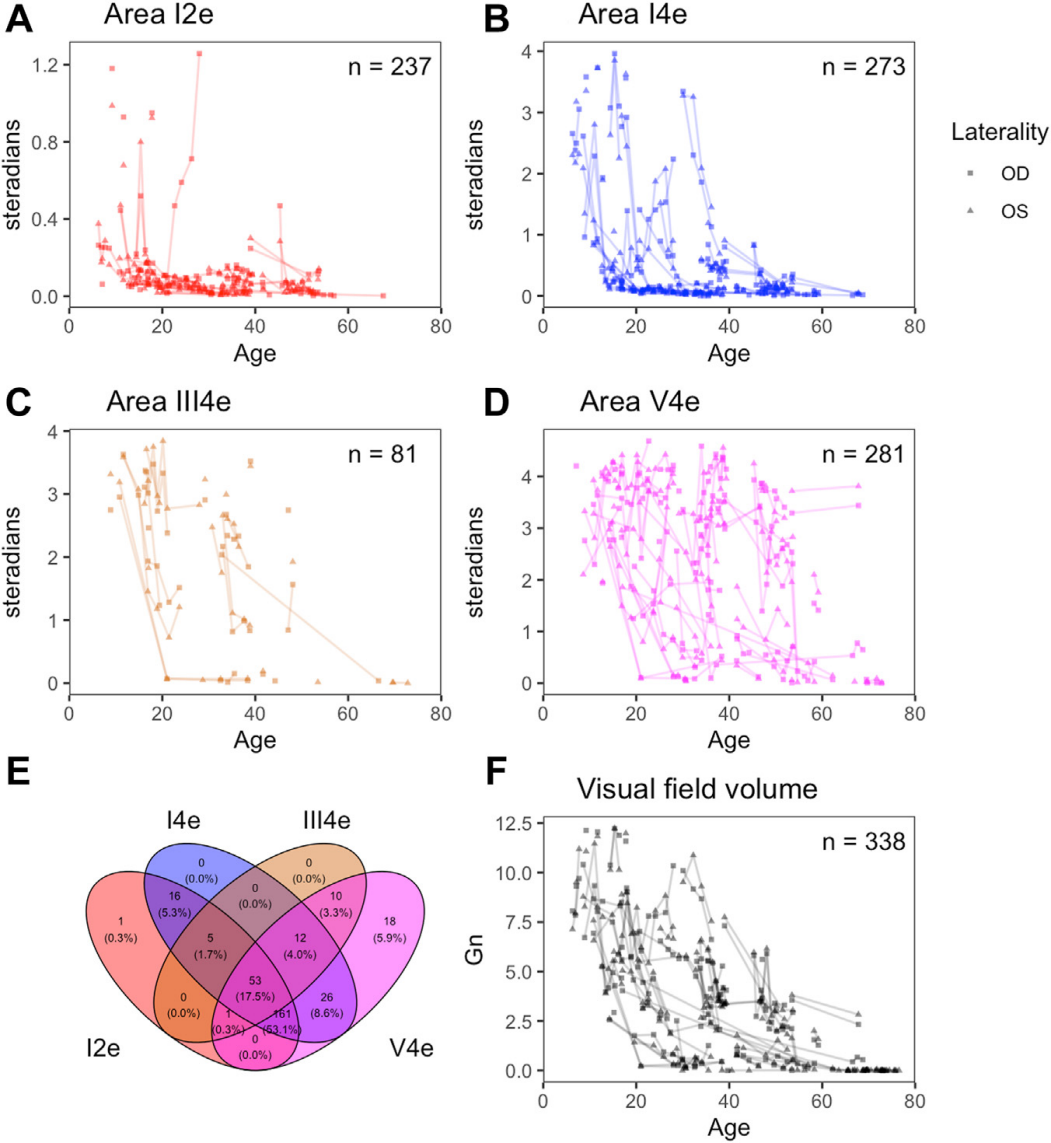
Volume shows wider dynamic range and greater total coverage of fields than individual isopters. **A,** Area of the I2e, (**B**) I4e, (**C**) III4e, and (**D**) V4e isopters plotted as a function of time. **E,** Venn diagram showing the number of fields with different combinations of isopters. **F,** Visual field volume as a function of age. The number of fields in each subpanel is designated by n. OD = right eye; OS = left eye.

Prior studies have suggested using the area of a single isopter to track the progression of choroideremia.^35^ This method benefits from simplicity but fails to fully capture the extent of disease. Smaller and dimmer targets (Fig 6A, B) lose area early and fail to capture late disease, and the larger or brighter targets (Fig 6C, D) do not detect early disease. Even in the face of missing data (Fig 6E), by incorporating data from all isopters, visual field volume (Fig 6F) can be used to track the entire course of the disease.

### Modeling Disease Progression Using Visual Field Volumes

We modeled the progression of vision loss in choroideremia using the visual field volume data. A hierarchical mixed-effects model was applied to visual field volume data for each patient in the cohort. The distributional assumptions of the naive model prior to fitting data are described in the Supplemental Methods and shown in Fig S7 (available at https://www.ophthalmologyscience.org). The joint effect of these priors is shown in Supplemental Methods Fig S8A (available at https://www.ophthalmologyscience.org). Fitting the model to the data reduced the range of plausible observations, as shown in Supplemental Methods, Fig S8B. Table 3 summarizes the parameter estimates for the model.

**Table 3:** Posterior Parameter Estimates for the Delayed Exponential Model with Hurdle

Parameter	Units	Mean	SD	Median	2.50%	97.50%
Volume at baseline	Gn	9.1	0.7	9.1	8.0	10.6
Delay	years	10.3	1.9	10.2	6.5	14.1
Delay (marginal of subject/eye)	years	12.6	9.1	10.4	2.7	36.4
Lambda		0.058	0.006	0.058	0.047	0.071
Lambda (marginal of subject)		0.068	0.045	0.058	0.019	0.188

Gn = Goldmann unit; SD = standard deviation.

Parameter estimates are derived by taking 2000 draws from the posterior distribution. The delay term describes the estimated age of onset. The lambda term indicates the proportion of volume lost per year after onset. As such, lambda is unitless. The parameters marked *marginal* are averaged over the uncertainty in the subjects or the eyes of subjects.

Inspecting the predictions for each subject illustrates how disease progression ranges widely across patients (Fig S9, available at https://www.ophthalmologyscience.org). The mixed-effects model accounts for the patient-specific variability in number of data points. Consequently, patients with more visits show less uncertainty around the mean than patients with fewer visits.

### Using the Model to Predict Prognosis

**Figure 10:**
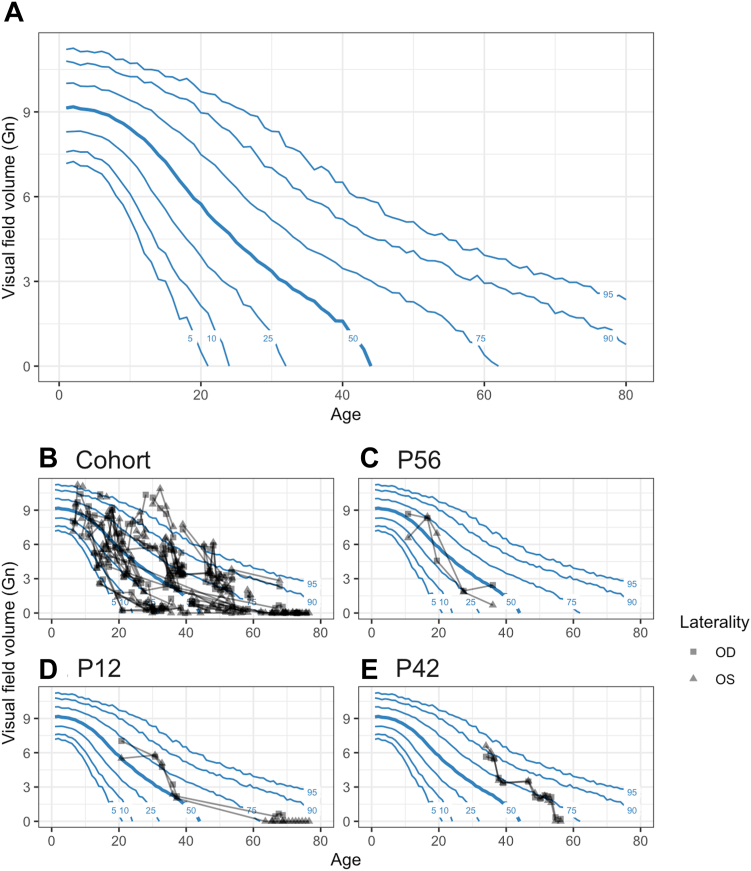
Progression chart for quantiles of the posterior predictions. **A,** Quantiles for predicting progression based on 2000 hypothetical patients drawn from the posterior distribution of the fitted statistical model. **B,** Observed visual field volume for all patients in the cohort. **C**–**E,** Observed visual field volumes for 3 highlighted patients. GN = Goldmann unit; OD = right eye; OS = left eye.

The posterior predictive interval can be used to gain insight into overall progression of choroideremia and as a practical tool for clinical care. In Figure 10A, we draw 2000 hypothetical patients from the posterior and show intervals with quantile markings from the 5th to 95th percentile. This yielded a representation akin to a pediatric growth chart—the visual field volume of patients can be expected to fall mostly within a quantile and to progress along that quantile (Fig 10C–E). Deviations from this may suggest disease modifiers or diagnostic uncertainty.

## Discussion

We report the use of volumetric analysis of Goldmann kinetic perimetry data to track disease progression in patients with choroideremia. This novel approach enables objective quantification of the visual field with demonstrable progression over a wide range of ages and genotypes and with minimal intereye variability. Furthermore, our approach displayed robustness against complex visual fields in patients with choroideremia and to variations in collection protocols inherent to retrospective cohorts. Additionally, this method can be readily applied to historical Goldmann perimetry data worldwide to enable longitudinal studies of disease progression.

It has previously been recognized that central VA does not provide a good assessment of disease stage in young patients with choroideremia.^35^ Our results and those of several previous studies have shown that VA in patients with choroideremia remains relatively stable until age 40 and then undergoes a sudden loss as the degeneration hits the fovea.^11^^,^^36^^,^^37^ Goldmann kinetic perimetry, and in particular the I4e isopter, has been used to demonstrate early decline in visual function in patients with choroideremia who had normal central acuity.^35^ However, the central I4e isopter is largely extinguished before age 30 and is thus less useful at older ages.^35^ The larger isopters are highly variable but show a fairly steep decline until age 60.^35^ Our findings demonstrate that volumetric analysis of complete Goldmann fields enables monitoring of disease progression over a wider age range and that the measurements from the right and left eyes were highly correlated. Thus, the volume of the GVF is a better metric for tracking disease progression in choroideremia than either VA or area of a single GVF isopter.

Microperimetry, which assesses subjective retinal sensitivity of the macula up to 45° either side of fixation,^38^ has been used as an outcome measure for choroideremia gene therapy trials.^39^ This outcome measure was considered reasonable because it assesses the macular area corresponding to the subretinal bleb where the gene therapy vector was delivered. However, the results from the 2014 United Kingdom-based trial were disappointing, with microperimetry thresholds declining at statistically similar rates in both treated and untreated eyes over the 2-year trial period.^40^ A related Canadian trial that used the same Aden-associated virus type 2 (AAV2)-based *CHM* gene therapy vector did not detect changes in microperimetry findings or VA over another 2-year trial period,^41^ suggesting that the therapy preserved but did not improve central vision. Five-year data from the original United Kingdom study identified a stable shift in the macular fixation locus from an untreated to a treated area of retina in 1 patient, although this possible treatment effect was not observed in any of the remaining trial participants.^42^

Recent work by Josan et al^43^ utilized volumetric interpolation of microperimetry data to demonstrate gains in macular sensitivity following gene therapy for *RPGR*-related X-linked retinitis pigmentosa. Their microperimetry volume tool enabled improved detection of macular function compared to the standard mean sensitivity measurement, which tended toward a zero value when limited microperimetry testing points were available.^43^^,^^44^ Despite this promising result and the attractiveness of microperimetry as a method that is potentially capable of specifically measuring only the treated area of retina, the approach may still be limited by the highly variable rates of disease progression between patients.

Our approach for visual field analysis has potential utility beyond clinical trial planning. The Goldmann perimeter has been essentially unchanged and is in clinical use from 1945^45^ until present, a period of almost 80 years. It is hard to imagine that any computerized perimeter or ophthalmic instrument will ever again approach this sort of longevity. As such, there remains a wealth of historical perimetry data in ophthalmology clinics worldwide that have yet to be quantitatively analyzed that will unlikely be replicated with modern instruments. The public availability of our volumetric analysis platform is intended to facilitate a reanalysis of these historical data, especially for long-term longitudinal studies requiring decades of follow-up.

We acknowledge several important limitations to this study. Our cohort of patients is modest in size. Patients were seen at The University of Iowa from 1966 until 2023, and protocols for assessing acuity and visual fields varied over this period. This variability in protocols leads to missing or incomplete data, which is a hallmark of most retrospective evaluations, and is a potential source for bias. As has been done in past studies,^30^^,^^46^ our statistical model assumes that field volume declines exponentially after a period of normal vision, but other progression curves are possible—for instance, exponential decline from birth or a sigmoidal pattern of progression. Distinguishing between progression curves will require identifying and monitoring individuals with pathogenic variants in *CHM* prior to self-reported changes in vision. Another important limitation of the method described here is the requirement for manual tracing of digitized GVF data, which represents the most important rate-limiting step of our method other than performing Goldmann perimetry itself. Previous research indicates that manual tracing of digitized GVF data is generally reliable even with minimally trained digitizers.^25^ However, the digitization step of our method would be largely unnecessary with the use of digital kinetic perimetry such as the Octopus perimeter.^47^ While our current manual workflow can be applied to kinetic fields collected on the Octopus 900 (Haag-Streit), we are currently developing software to directly import these. This will allow longitudinal comparisons to continue as clinics transition from the out-of-production Goldmann perimeters to Octopus perimetry.

In summary, we have presented a new method of visual field analysis using historical Goldmann kinetic perimetry data to determine the long-term progression of choroideremia. This method demonstrated that, unlike VA, the visual field in choroideremia declines at a relatively constant rate over time and has remarkable consistency between eyes for an affected individual. These findings have broad implications for the rational development of therapeutic outcome measures in choroideremia. The method is also broadly applicable to other eye diseases that affect the visual field and enables the utilization of legacy visual field data for clinical research.
