# Supplementary material for: Using Goldmann Visual Field Volume to Track Disease Progression in Choroideremia
Source: Ophthalmol Sci. 2023 Sep 14;3(4):100397. doi: 10.1016/j.xops.2023.100397 (PMC10630671; doi:10.1016/j.xops.2023.100397)
Supplement: Table S2 [file mmc1.pdf]

FAM	IND	DBSNP	HGVS g.	HGVS c.	HGVS p.
F1	P1		Breakpoint Not Determined		
F1	P2		Breakpoint Not Determined		
F2	P3		Breakpoint Not Determined		
F3	P4		Breakpoint Not Determined		
F4	P5		Breakpoint Not Determined		
F5	P6		Breakpoint Not Determined		
F6	P7		NC_000023.10:g.[85034854_85113182delinsCATTTGCAATTGAG;85113183_85121844inv;85121845_85122135del]		
F7	P8		NC_000023.10:g.[85166301del;85166305del]	NC_000023.10(NM_000390.4):c.[1205del;1209del]	NC_000023.10(NP_000381.1):p.(His402Lysfs*14)
F8	P9		NC_000023.10:g.84539284_86055979delinsTGCCCCA		
F9	P10		NC_000023.10:g.84790935_87522740delinsTATA		
F9	P11		NC_000023.10:g.84790935_87522740delinsTATA		
F10	P12		NC_000023.10:g.85100378_85626665del		
F11	P13		NC_000023.10:g.85100822_85178695delinsATAATGAACAATAATGAATAATGAGATTGAATA	NC_000023.10(NM_000390.4):c.1167-12352_*18813delinsTATTCAATCTCATTATTCATTATTGTTCATTAT	
F12	P14	rs587776746	NC_000023.10:g.85133997_85134000del	NM_000390.4:c.1584_1587del	NM_000390.4(NP_000381.1):p.(Val529Hisfs*7)
F13	P15	rs587776746	NC_000023.10:g.85133997_85134000del	NM_000390.4:c.1584_1587del	NM_000390.4(NP_000381.1):p.(Val529Hisfs*7)
F14	P16		NC_000023.10:g.85145879_85152933del	NC_000023.10(NM_000390.4):c.1413+2720_1510+3316del	NC_000023.10(NP_000381.1):p.(Ser473Trpfs*4)
F14	P17		NC_000023.10:g.85145879_85152933del	NC_000023.10(NM_000390.4):c.1413+2720_1510+3316del	NC_000023.10(NP_000381.1):p.(Ser473Trpfs*4)
F15	P18	rs1926208868	NC_000023.10:g.85155705_85155706delinsC	NM_000390.4:c.1358_1359delinsG	NM_000390.4(NP_000381.1):p.(Ser453*)
F16	P19	rs1057517716	NC_000023.10:g.85156096G>A	NM_000390.4:c.1342C>T	NM_000390.4:p.(Gln448*)
F17	P20	rs1057520629	NC_000023.10:g.85156104G>C	NM_000390.4:c.1334C>G	NM_000390.4(NP_000381.1):p.(Ser445*)
F18	P21		NC_000023.10:g.85203106_85546828del	NC_000023.10(NM_000390.4):c.-244286_1166+8058del	
F18	P22		NC_000023.10:g.85203106_85546828del	NC_000023.10(NM_000390.4):c.-244286_1166+8058del	
F19	P23		NC_000023.10:g.85204844_85215309dup	NC_000023.10(NM_000390.4):c.703-1325_1166+6316dup	
F19	P24		NC_000023.10:g.85204844_85215309dup	NC_000023.10(NM_000390.4):c.703-1325_1166+6316dup	
F20	P25	rs1929999634	NC_000023.10:g.85211229_85211230del	NM_000390.4:c.1094_1095del	NM_000390.4(NP_000381.1):p.(Leu365Argfs*52)
F21	P26		NC_000023.10:g.85212902dup	NM_000390.4:c.898dup	NM_000390.4(NP_000381.1):p.(Thr300Asnfs*7)
F22	P27	rs132630266	NC_000023.10:g.85212923G>A	NM_000390.4:c.877C>T	NM_000390.4(NP_000381.1):p.(Arg293*)
F23	P28	rs1556307648	NC_000023.10:g.85212933_85212934dup	NM_000390.4:c.866_867dup	NM_000390.4(NP_000381.1):p.(Val290Trpfs*2)
F24	P29		NC_000023.10:g.85213915_85213916del	NM_000390.4:c.770_771del	NM_000390.4(NP_000381.1):p.(Phe257*)
F25	P30	rs886041178	NC_000023.10:g.85213928G>A	NM_000390.4:c.757C>T	NM_000390.4(NP_000381.1):p.(Arg253*)
F26	P31	rs886041178	NC_000023.10:g.85213928G>A	NM_000390.4:c.757C>T	NM_000390.4(NP_000381.1):p.(Arg253*)
F27	P32	rs886041178	NC_000023.10:g.85213928G>A	NM_000390.4:c.757C>T	NM_000390.4(NP_000381.1):p.(Arg253*)
F27	P33	rs886041178	NC_000023.10:g.85213928G>A	NM_000390.4:c.757C>T	NM_000390.4(NP_000381.1):p.(Arg253*)
F28	P34	rs886041178	NC_000023.10:g.85213928G>A	NM_000390.4:c.757C>T	NM_000390.4(NP_000381.1):p.(Arg253*)
F28	P35	rs886041178	NC_000023.10:g.85213928G>A	NM_000390.4:c.757C>T	NM_000390.4(NP_000381.1):p.(Arg253*)
F28	P36	rs886041178	NC_000023.10:g.85213928G>A	NM_000390.4:c.757C>T	NM_000390.4(NP_000381.1):p.(Arg253*)
F28	P37	rs886041178	NC_000023.10:g.85213928G>A	NM_000390.4:c.757C>T	NM_000390.4(NP_000381.1):p.(Arg253*)
F28	P38	rs886041178	NC_000023.10:g.85213928G>A	NM_000390.4:c.757C>T	NM_000390.4(NP_000381.1):p.(Arg253*)
F28	P39	rs886041178	NC_000023.10:g.85213928G>A	NM_000390.4:c.757C>T	NM_000390.4(NP_000381.1):p.(Arg253*)
F28	P40	rs886041178	NC_000023.10:g.85213928G>A	NM_000390.4:c.757C>T	NM_000390.4(NP_000381.1):p.(Arg253*)
F29	P41	rs886041178	NC_000023.10:g.85213928G>A	NM_000390.4:c.757C>T	NM_000390.4(NP_000381.1):p.(Arg253*)
F30	P42	rs886041178	NC_000023.10:g.85213928G>A	NM_000390.4:c.757C>T	NM_000390.4(NP_000381.1):p.(Arg253*)
F31	P43	rs886041178	NC_000023.10:g.85213928G>A	NM_000390.4:c.757C>T	NM_000390.4(NP_000381.1):p.(Arg253*)
F32	P44	rs776256380	NC_000023.10:g.85213970G>A	NM_000390.4:c.715C>T	NM_000390.4(NP_000381.1):p.(Arg239*)
F33	P45	rs886043716	NC_000023.10:g.85218725_85218728del	NC_000023.10(NM_000390.4):c.649_652del	NC_000023.10(NP_000381.1):p.(Tyr217Hisfs*14)
F34	P46	rs886041177	NC_000023.10:g.85218846_85218847del	NM_000390.4:c.525_526del	NM_000390.4(NP_000381.1):p.(Glu177Lysfs*6)
F35	P47	rs886041177	NC_000023.10:g.85218846_85218847del	NM_000390.4:c.525_526del	NM_000390.4(NP_000381.1):p.(Glu177Lysfs*6)
F35	P48	rs886041177	NC_000023.10:g.85218846_85218847del	NM_000390.4:c.525_526del	NM_000390.4(NP_000381.1):p.(Glu177Lysfs*6)
F36	P49	rs886041176	NC_000023.10:g.85219056_85219059del	NC_000023.10(NM_000390.4):c.315_318del	
F36	P50	rs886041176	NC_000023.10:g.85219056_85219059del	NC_000023.10(NM_000390.4):c.315_318del	
F36	P51	rs886041176	NC_000023.10:g.85219056_85219059del	NC_000023.10(NM_000390.4):c.315_318del	
F37	P52		NC_000023.10:g.85219056G>A	NM_000390.4:c.316C>T	NM_000390.4:p.(Gln106*)
F38	P53		NC_000023.10:g.85267479_85267494delins[GGCCGGGCGCGGTGGCTCACGCCTGTAATCCC;NC_000003.10:g.121534573_121746886;TGGAAGTTAAGTTAGTTACT]		
F39	P54	rs786204761	NC_000023.10:g.85282494C>T	NC_000023.10(NM_000390.4):c.116+1G>A	
F40	P55	rs886041174	NC_000023.10:g.85302487C>A	NC_000023.10(NM_000390.4):c.49+1G>T	
F41	P56		NC_000023.10:g.85302505_85302516del	NM_000390.4:c.22_33del	NM_000390.4(NP_000381.1):p.(Glu8_Val11del)

Supplemental Table 2. HGVS specification for *CHM* genotypes. Variations are annotated with dbSNP identifiers and full HGVS-compliant formats, including the genomic and transcriptomic coordinates, as appropriate.
