# Supplementary material for: Using Goldmann Visual Field Volume to Track Disease Progression in Choroideremia
Source: Ophthalmol Sci. 2023 Sep 14;3(4):100397. doi: 10.1016/j.xops.2023.100397 (PMC10630671; doi:10.1016/j.xops.2023.100397)
Supplement: Figure S9 [file mmc2.pdf]

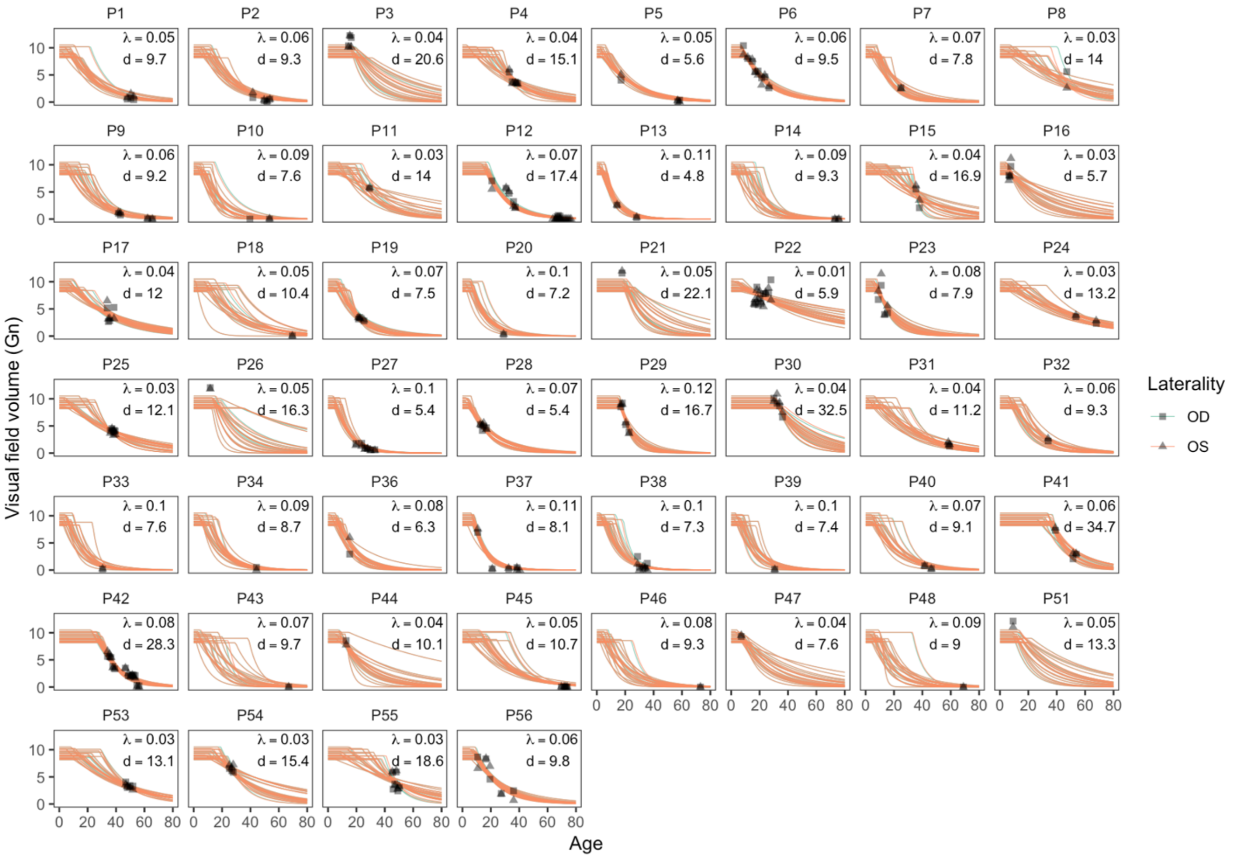

Supplemental Figure 9. Posterior predictions of disease progression for each eye in the dataset. The lines indicate the predictions of the patient's course of disease assuming the delayed exponential model. Twenty lines are shown for each eye. The median rate constant, λ, and median delay, d, are computed based on 1,000 draws from the posterior distribution for each eye of each individual.
