# Supplementary material for: Using Goldmann Visual Field Volume to Track Disease Progression in Choroideremia
Source: Ophthalmol Sci. 2023 Sep 14;3(4):100397. doi: 10.1016/j.xops.2023.100397 (PMC10630671; doi:10.1016/j.xops.2023.100397)
Supplement: Supplemental Methods [file mmc3.pdf]

Supplemental Methods

Component 1 (Delayed Exponential)

Volumes greater than 0 were considered to arise from a normal distribution, with a mean value equal to a delayed exponential curve. This model assumes that visual field volume remains normal until some time after birth, at which point volume follows an exponential decline. (The delay term we estimate is not equivalent to self-reported age at first visual symptom. Self-reported age of first visual symptoms is unreliable in slowly progressing, peripheral disease.) The key parameters are volume at baseline (V_0), the delay between birth and onset (d), and the rate of volume loss (λ). We assumed that (1) everyone in the cohort is born with approximately normal volume, (2) that the median delay is about 10 years after birth, and (3) the rate of decline is negative. We allowed the rate of decline term to vary by individual and the delay term to vary by individual and by eye. The model takes the graphical form shown in **SM Figure 7A**.

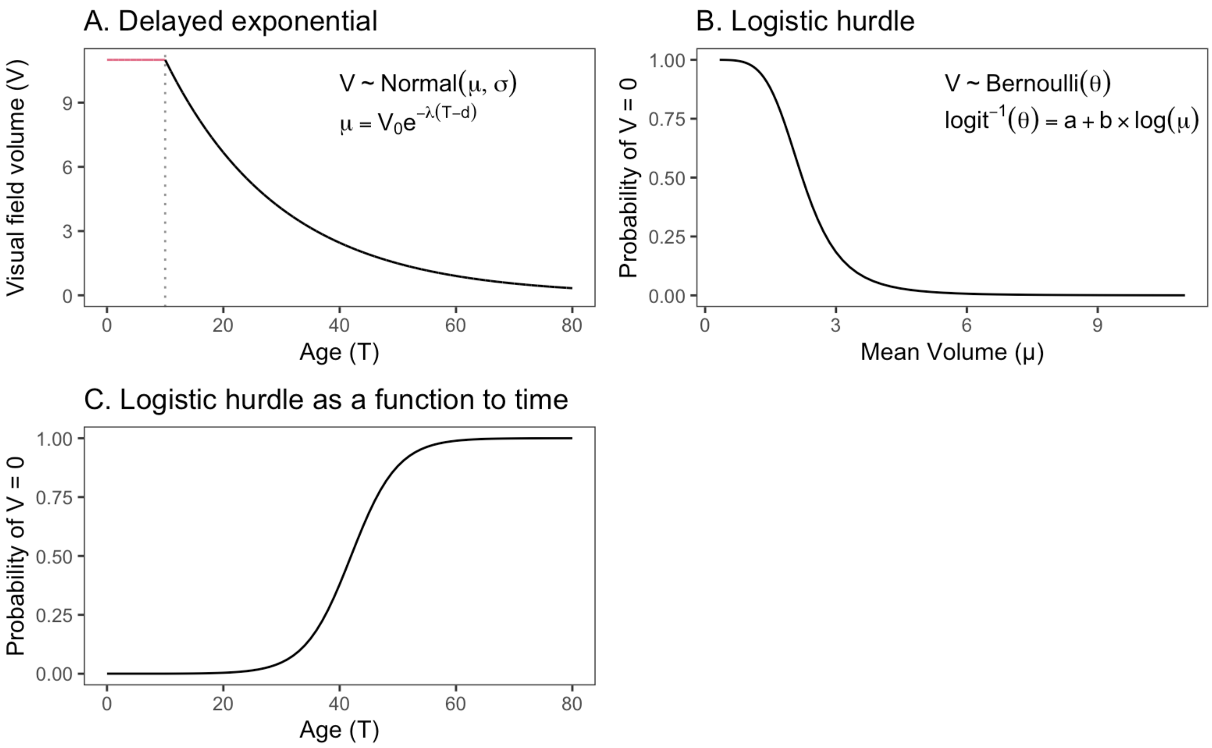

SM Figure 7. Components of the model for an idealized example. (**A**) The delayed exponential function assumes that the mean visual field volume (μ) starts approximately normal (V_0), then after a delay (d, shown in red), begins to decrease at an exponential rate (λ). (**B**) The logistic hurdle assumes that the probability (θ) of recording a visual field volume of 0 Goldmanns is a function of the mean volume (μ). (People with poor vision have trouble performing the test.) (**C**) Since the mean visual field volume depends on age (*T*), the probability (θ) of observing a visual field volume of 0 is also a function of age.

Equations: When the observed GVF volume is greater than zero ($V > 0$), the model approximates a delayed (shifted) exponential function[1]:

$$V \sim \text{Normal}(\mu, \sigma)$$

$$\mu = V_0 \times \exp\left(-\lambda \times (T - d)\right)$$

$$\lambda = \lambda_{CHM} \times \exp\left(z_{1[S]}\sigma_1\right)$$

$$d = d_{CHM} \times \exp\left(z_{2[S]}\sigma_2\right) \times \exp\left(z_{3[SE]}\sigma_3\right)$$

where *S* is an index variable for each subject and *SE* is an index variable for each eye within each subject. Priors are as follows:

$$V_0 \sim \text{LogNormal}(2.42, 0.11)$$

$$d_{CHM} \sim \text{LogNormal}\left(\log(10.0), \log(1.80)\right)$$

$$\lambda_{CHM} \sim \text{Beta}(0.4, 2.76)$$

$$\sigma \sim \text{Exponential}(1)$$

$$\sigma_1 \sim \text{Exponential}(5)$$

$$\sigma_2 \sim \text{Exponential}(10)$$

$$\sigma_3 \sim \text{Exponential}(1)$$

$$z_1, z_2, z_3 \sim \text{Normal}(0, 1)$$

Mixed effects: We included effects for each subject (*S*) for λ, and we included effects for subject (*S*) and eye within subject (*SE*) for d. We coded these effects as exponentiated z-distributions (i.e., non-centered Log-Normal distributions) to maintain strictly positive estimates for λ and d.

Priors: The prior for GVF volume at baseline, V_0, was estimated by fitting a Log-Normal distribution to GVF volumes calculated from a published set of normal children (age 13-15).[2] Briefly, we assumed that the 95% confidence intervals reported described a Normal distribution. We traced and calculated volumes for the isopters for the lower and upper bounds. From the lower and upper bounds, we computed the mean and standard deviation. Using these parameters, we generated random 10,000 values, then fit a Log-

Normal distribution to these values. The log(mean) and log(standard deviation) were used as estimates for the prior distribution. The prior for the delay in onset, d_{CHM}, was estimated from the reported average age of onset of 10 years. The prior for the rate of volume loss, λ_{CHM} , was chosen to place the probability density near the estimate of 0.125 for the rate at which residual autofluorescence area was lost per year [3]. The Beta distribution conveniently bounds λ_{CHM} between 0 and 1. The priors for standard deviations (σ, σ_1, σ_2, and σ_3) where chosen to constrain the joint effect of the priors to within plausible ranges[4].

Component 2 (Logistic Hurdle)

For volumes equal to 0, volumes were considered to arise from a Bernouli distribution. The Bernouli distribution is like a biased coin toss. In this case, the bias is determined by the mean visual field volume predicted by Component 1. That is, as the mean visual field volume approaches 0, the chance of observing a 0 value increases (e.g., the test becomes more difficult to perform or isn't performed at all). This second component allowed us to maintain the error properties of the normal distribution while accounting for the fact that volumes can never be measured less than 0. The model takes the graphical form shown in **SM Figure 7B**.

Equations: Thus, when the observed GVF volume is 0 ($V = 0$), V arises from a Bernoulli distribution:

$$V \sim Bernoulli(\theta)$$
$$logit(\theta) = a + b \times \mu$$

with priors:

$$a \sim Normal(4, 1)$$

$$b \sim Normal(-5, 1)$$

The logistic equation makes the chance of observing zero depend on the predicted volume: tiny mean volumes will be more likely to result in an observed volume of 0 than will larger mean volumes.

Fitting the model

The distributional assumptions of the naive model prior to fitting data are represented in **SM Figure 8A**. Fitting the model to the data reduced the range of plausible observations, shown in **SM Figure 8B**.

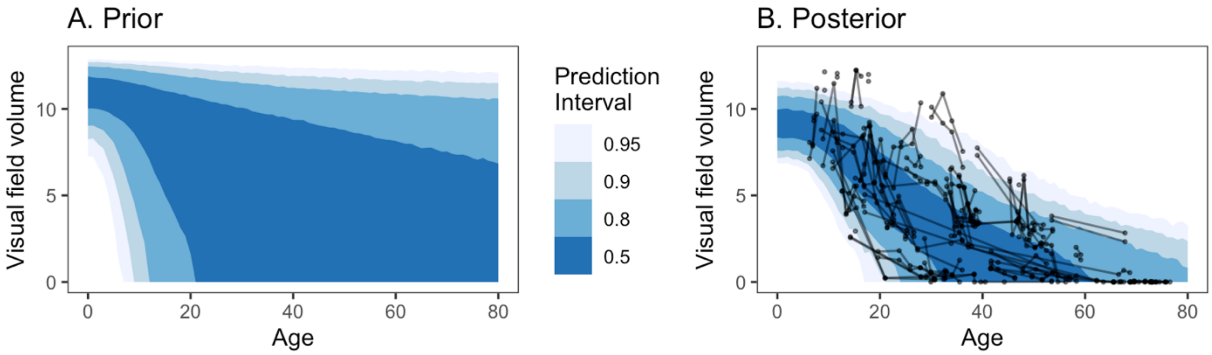

SM Figure 8. Predictive intervals before and after fitting the model to the data. (**A**) 50%, 80%, 90%, and 95% prior predictive intervals marginalized over the variability in possible subjects. (**B**) 50%, 80%, 90%, and 95% posterior predictive intervals marginalized over the variability in possible subjects, after fitting the model to the data.

References

1. Clarke G, Collins RA, Leavitt BR, et al. A one-hit model of cell death in inherited neuronal degenerations. Nature 2000;406(6792):195-9.
2. Patel DE, Cumberland PM, Walters BC, et al. Study of Optimal Perimetric Testing In Children (OPTIC): Normative Visual Field Values in Children. Ophthalmology 2015;122(8):1711-7.
3. Aylward JW, Xue K, Patricio MI, et al. Retinal Degeneration in Choroideremia follows an Exponential Decay Function. Ophthalmology 2018;125(7):1122-4.
4. Wesner JS, Pomeranz JPF. Choosing priors in Bayesian ecological models by simulating from the prior predictive distribution. Ecosphere 2021;12(9 , ISSN = 2150-8925 2150-8925 , DOI = 10.1002/ecs2.3739).
